# Expression of Aromatase in Radial Glial Cells in the Brain of the Japanese Eel Provides Insight into the Evolution of the *cyp191a* Gene in Actinopterygians

**DOI:** 10.1371/journal.pone.0044750

**Published:** 2012-09-05

**Authors:** Shan-Ru Jeng, Wen-Shiun Yueh, Yi-Ting Pen, Marie-Madeleine Gueguen, Jérémy Pasquier, Sylvie Dufour, Ching-Fong Chang, Olivier Kah

**Affiliations:** 1 Department of Aquaculture, National Kaohsiung Marine University, Kaohsiung, Taiwan; 2 Team NEED, Institut de Recherche en Santé, Environnement et Travail, INSERM U1085, IFR140, Université de Rennes 1, Rennes, France; 3 Research Unit BOREA, Biology of Aquatic Organisms and Ecosystems, CNRS 7208/IRD 207/UPMC, Muséum National d'Histoire Naturelle, Paris, France; 4 Department of Aquaculture, Center of Excellence for Marine Bioenvironment and Biotechnology, National Taiwan Ocean University, Keelung, Taiwan; University of Hyderabad, India

## Abstract

The *cyp19a1* gene that encodes aromatase, the only enzyme permitting conversion of C19 aromatizable androgens into estrogens, is present as a single copy in the genome of most vertebrate species, except in teleosts in which it has been duplicated. This study aimed at investigating the brain expression of a *cyp19a1* gene expressed in both gonad and brain of Japanese eel, a basal teleost. By means of immunohistochemistry and in situ hybridization, we show that *cyp19a1* is expressed only in radial glial cells of the brain and in pituitary cells. Treatments with salmon pituitary homogenates (female) or human chorionic gonadotrophin (male), known to turn on steroid production in immature eels, strongly stimulated *cyp19a1* messenger and protein expression in radial glial cells and pituitary cells. Using double staining studies, we also showed that aromatase-expressing radial glial cells exhibit proliferative activity in both the brain and the pituitary. Altogether, these data indicate that brain and pituitary expression of Japanese eel *cyp19a1* exhibits characteristics similar to those reported for the brain specific *cyp19a1b* gene in teleosts having duplicated cyp19a1 genes. This supports the hypothesis that, despite the fact that eels also underwent the teleost specific genome duplication, they have a single *cyp19a1* expressed in both brain and gonad. Such data also suggest that the intriguing features of brain aromatase expression in teleost fishes were not gained after the whole genome duplication and may reflect properties of the *cyp19a1* gene of ancestral Actinopterygians.

## Introduction

In his famous book “Evolution by Gene Duplication”, Suzumu Ohno [1] suggested that the large size of the vertebrate genome is the result of whole genome duplications and that such events are major triggers of evolution. Since that time, Ohno's hypotheses have been largely confirmed and it is now accepted that two distinct genome duplication events, known as 1R and 2R, occurred early in vertebrate evolution prior to the fish-tetrapod split [2]. It is also believed that a third round of whole genome duplication, referred to as 3R, occurred soon after the emergence of teleost fishes [3], [4]. One of the evidences for this third event stems from the fact that fish have 7 or 8 hox genes while tetrapods have only 4 [5].

One of the genes that appear to have been duplicated in teleost fishes is the *cyp19a1* gene. In most vertebrates, *cyp19a1* that encodes aromatase, the only enzyme able to convert C19 aromatizable androgens into C18 estrogens [6]. As such aromatase plays crucial roles in reproductive and non-reproductive mechanisms in vertebrates [7]. Under the control of alternative usage of different promoters, *cyp19a1* is expressed in multiple tissues, including the brain [8], [9]. Estrogens produced in the brain, sometimes referred to as neuroestrogens, exhibit neurotrophic and/or neuroprotective functions and are believed to exert strong influences on neuronal development, survival and plasticity according to complex and still partially uncovered mechanisms [10]–[12].

While most vertebrates express *cyp19a1* in the brain through usage of brain specific promoters [13], teleost fishes are unique in having two *cyp19a1*genes [14]. These two genes, named *cyp19a1a* and *cyp19a1b,* encode different aromatases, aromatase A and aromatase B, respectively [15], [16]. These genes exhibit a marked tissue-specificity of expression, *cyp19a1a* being expressed mainly in the gonads and *cyp19a1b* mainly expressed in the brain, suggesting a partition of functions of the original gene [17].

Aromatase expression and regulation in the brain of adult teleost fishes exhibits some particular features compared to tetrapods. First, many studies have documented the fact that the brain of teleost fish has exceptionally high aromatase activity due to the strong expression of the *cyp19a1b* gene [17], [18]. Second, this gene is only expressed in a unique brain cell type, the radial glial cells [17], [19]–[22]. Such cells act as progenitors during vertebrate embryonic development, but disappear at the end of the embryonic period in mammals in which they become astrocytes or the so-called B cells [23]. In non-mammalian vertebrates, and particularly in teleost fishes, radial glial cells persist in many brain regions and support the well-documented capacity of the brain to grow during adulthood [24]–[27]. Detailed studies in zebrafish [25], [26] and in pejerrey [22], have shown that radial glial cells, many of which express aromatase, keep their neurogenic properties and serve as neuronal progenitors during adult life. Third, *cyp19a1b* in teleost fish is strongly up-regulated by estrogens [21] and some androgens [28], and in some species such as the medaka it shows sxeula dimorphic expression [29]. This effect is mediated by estrogen receptor binding on an estrogen-responsive element located on the proximal promoter [17], [21], [28], [30], [31].

**Figure 1:**
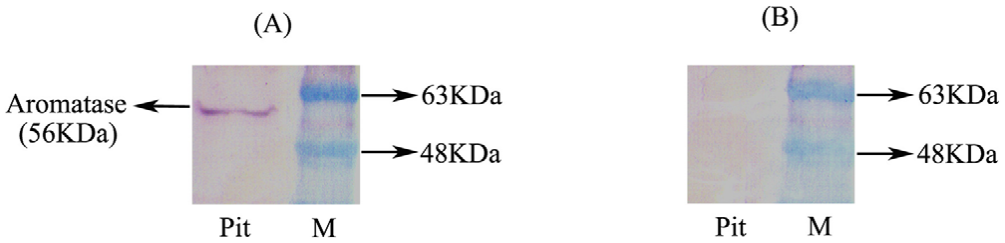
Western blotting analysis of aromatase expression in male pituitary extracts. (A) Incubation with the aromatase antibody diluted (1∶1000) yielded a single band at the expected size of 56 Kd. (B) Pre-absorption of the antiserum diluted 1∶1000 with the peptide NH2-EKDSE LTMMF TPRRR Q- COOH (25 μM) caused disappearance of the band.

**Figure 2:**
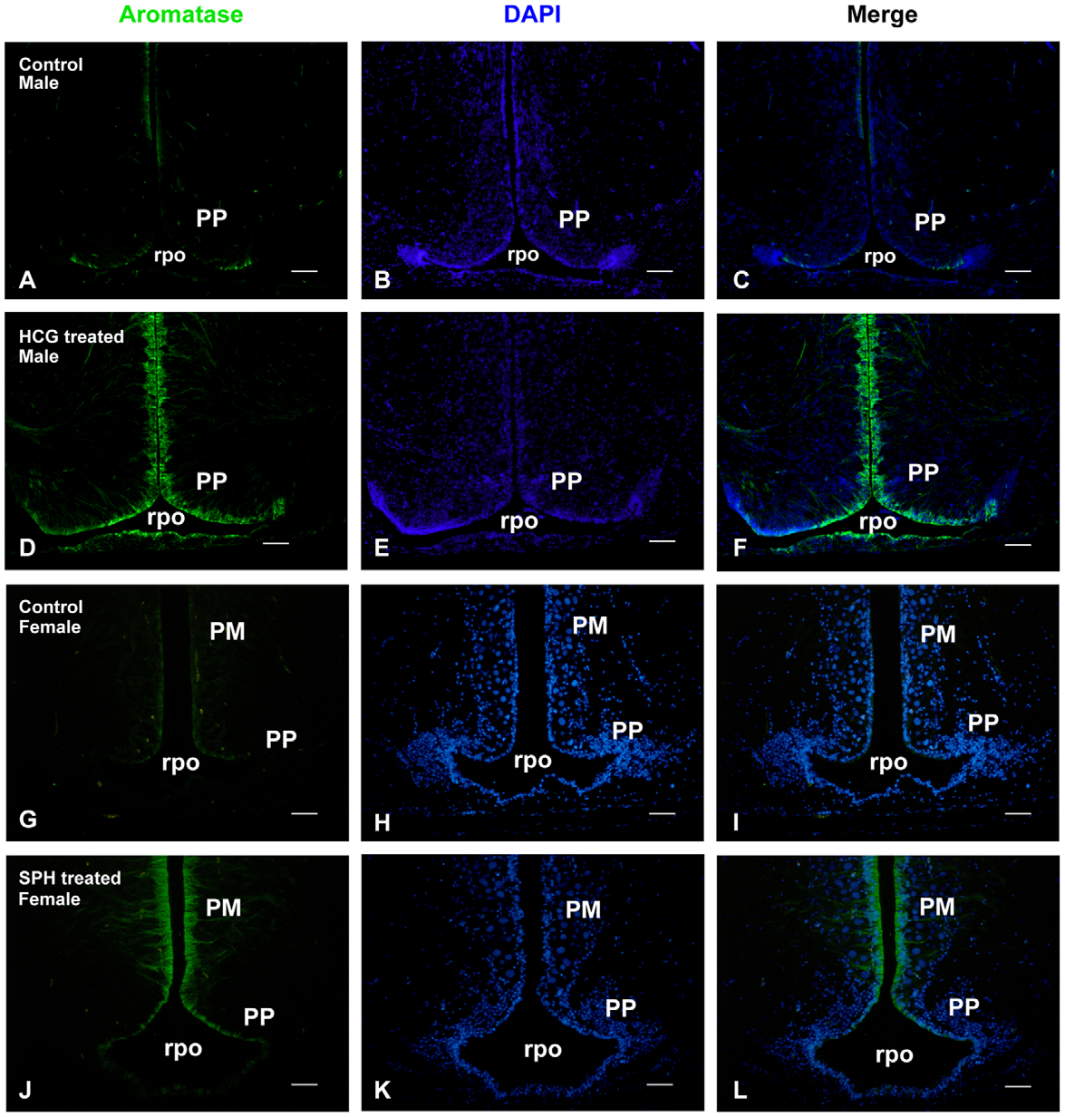
Hormonal treatment strongly increases aromatase immunoreactivity in the brain of Japanese eel. Transverse sections in the preoptic area of males (A–F) and females (G–L) showing aromatase-immunoreactivity in control animals (males: A–C; females G–I) and animals treated with either human chorionic gonadotrophin (HCG, males: D–F) or salmon pituitary homogenates (SPH, females: J–L). One can see that hormonal treatment strongly increases the aromatase immunoreactivity in cells bordering the preoptic recess (rpo). Pictures in A and D (or G and J) were taken with the same exposure time to allow comparison. PP: parvocellular preoptic nucleus; PM: magnocellular preoptic nucleus. A–F: Bar  = 75 μm; G–L: Bar  = 50 μm.

**Figure 3:**
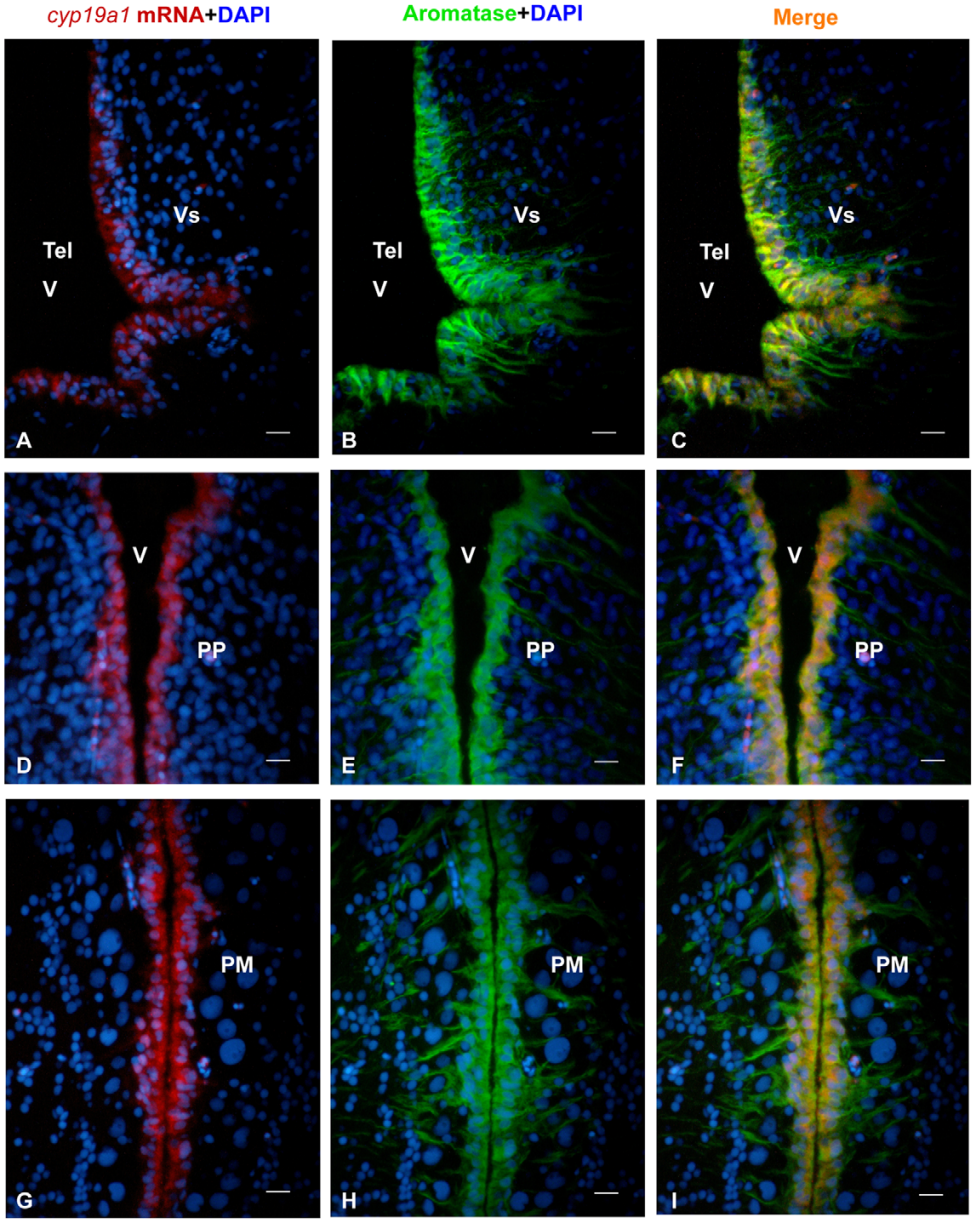
Distribution of *cyp19a1b* mRNA and aromatase protein in the brain of the Japanese eel. A, D, G: *Cyp19a1b* mRNA in the brain of the Japanese eel as revealed by *in situ* hybridization in the supracommissural nucleus of the subpallium (Vs), the parvocellular preoptic nucleus (PP) and the magnocellular preoptic nucleus (PM). Note that the signal is consistently restricted to the regions adjacent to the ventricles. B, E, H: Aromatase protein in the brain of the Japanese eel as revealed by immunohistochemistry on the same sections than in A, D and G. One can see that immunoreactive cells have their nuclei along the ventricles and long lateral processes C, F, I: Merges showing overlapping (yellow color) of *cyp19a1b* mRNA and aromatase protein in the brain of the Japanese eel as revealed by immunohistochemistry on the same sections than in A, D and G. Only the radial processes do not show co-expression. All bars  = 20 μm.

**Figure 4:**
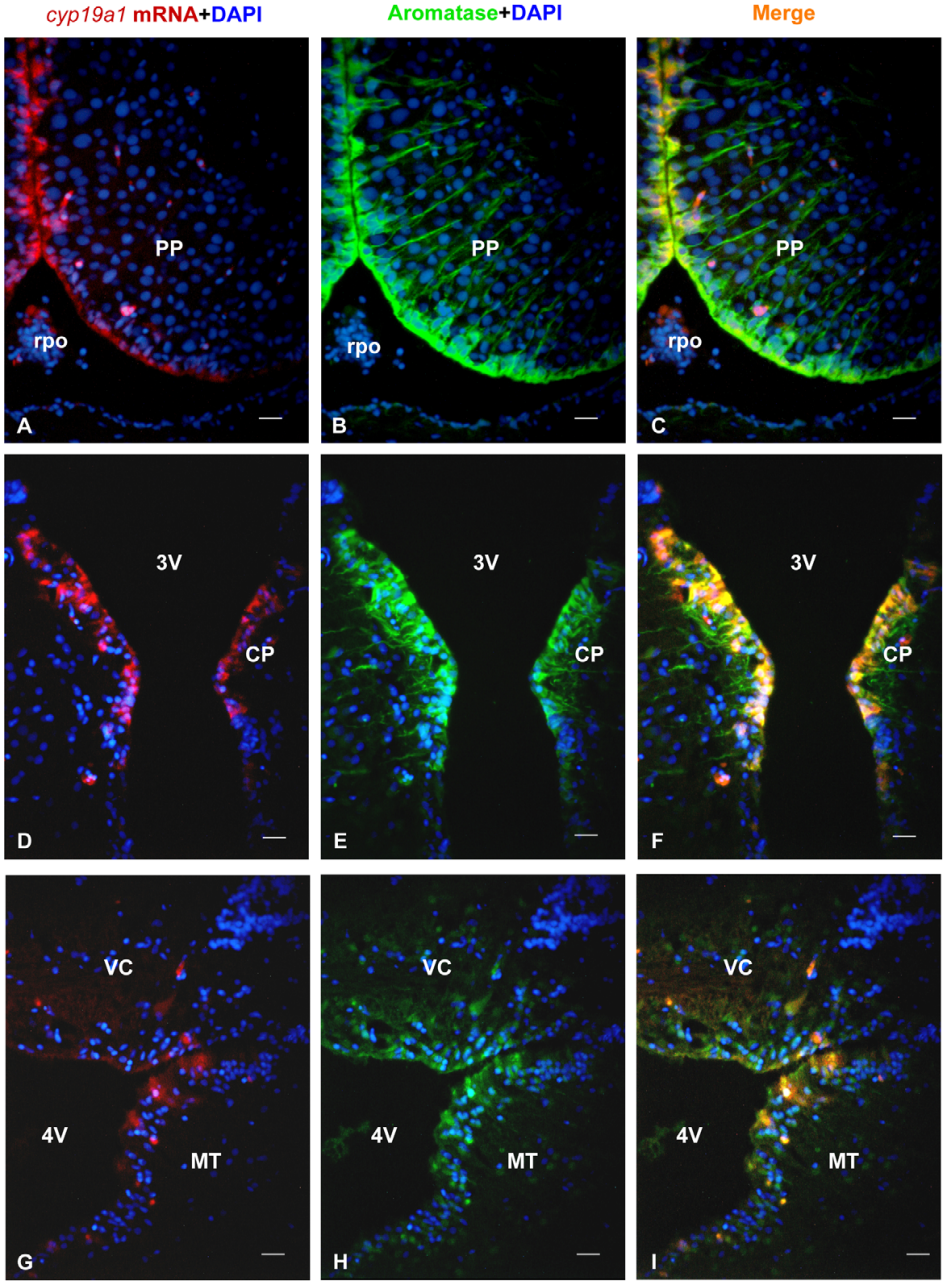
Distribution of *cyp19a1b* mRNA and aromatase protein in the brain of the Japanese eel. A, D, G: *Cyp19a1b* mRNA in the brain of the Japanese eel as revealed by *in situ* hybridization in the supracommissural nucleus of the subpallium (Vs), the dorsal central thalamic nucleus (CP) and around the 4^th^ ventricle (4V) between the valvula of the cerebellum (VC) and the midbrain tegmentum (MT). Note that the signal is consistently restricted to the regions adjacent to the ventricles. B, E, H: Aromatase protein in the brain of the Japanese eel as revealed by immunohistochemistry on the same sections than in A, D and G. One can see that immunoreactive cells have their nuclei along the ventricles and long lateral processes C, F, I: Merges showing overlapping (yellow color) of *cyp19a1b* mRNA and aromatase protein in the brain of the Japanese eel as revealed by immunohistochemistry on the same sections than in A, D and G. Only the radial processes do not show co-expression. All bars  = 20 μm.

**Figure 5:**
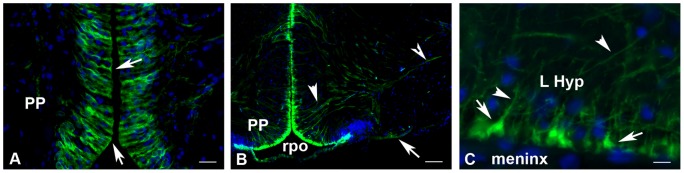
Details of the aromatase-positive cells characterizing them as radial glial cells. (A) Proximal processes (arrows) reaching the ventricle at the level of the anterior periventricular preoptic nucleus. Bar  = 15 μm (B) Long radial processes (arrowheads) some of which end at the ventral surface of the brain (arrow) while others project more laterally. Bar  = 75 μm (C) End-feet of the distal processes at the periphery of the lateral hypothalamus (L Hyp) close to the meninx. Bar  = 8 μm.

**Figure 6:**
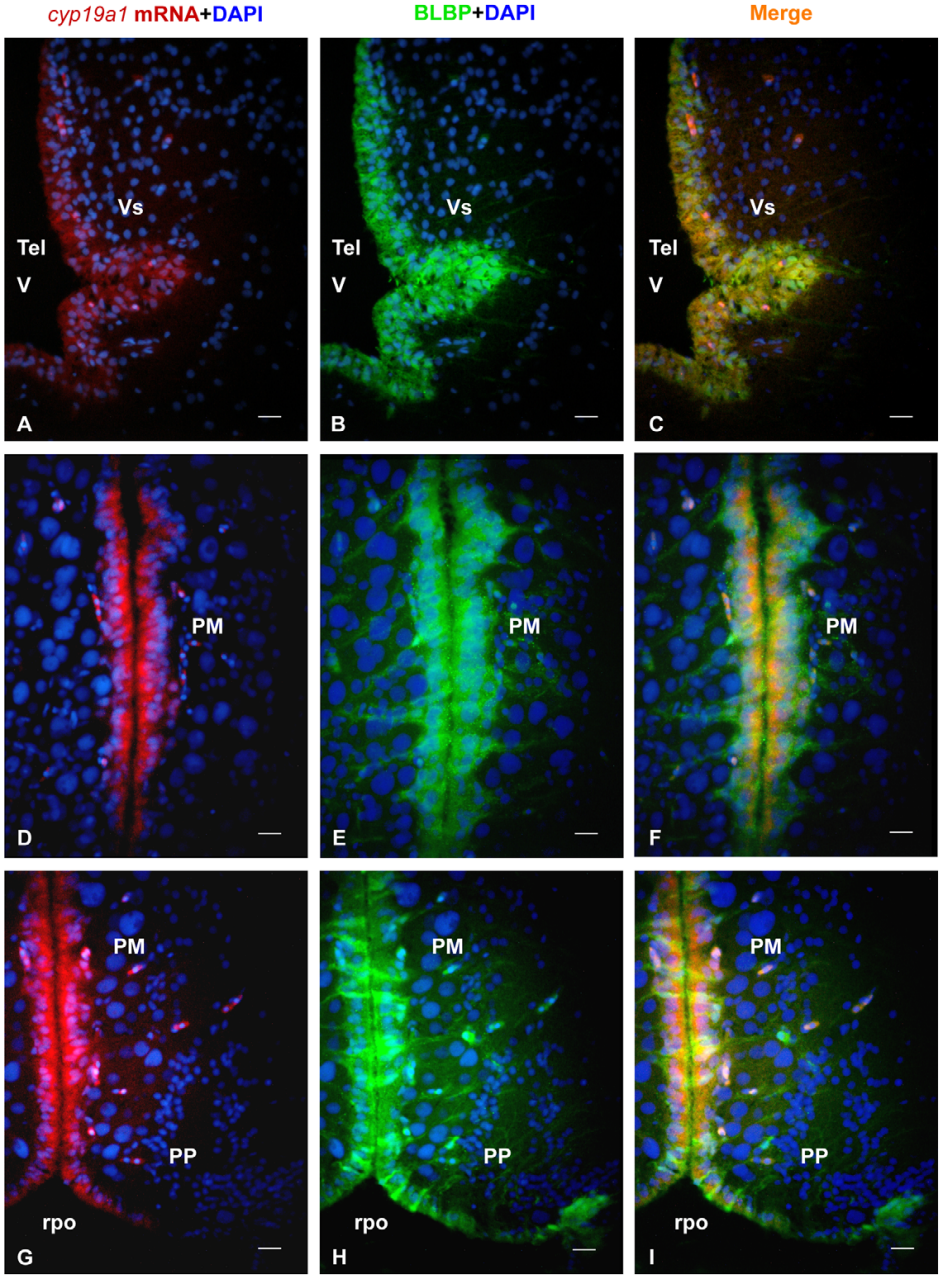
*In situ* hybridization of *cyp19a1b* mRNA combined to BLBP (Brain Lipid Binding Protein) immunohistochemistry confirms the radial glial nature of the aromatase-positive cells. A, D, G: *Cyp19a1b* mRNA in the brain of the Japanese eel as revealed by *in situ* hybridization in the post-commissural nucleus of the subpallium (Vs), the magnocellular preoptic nucleus (PM) and the parvocellular preoptic nucleus (PP). Note that the signal is consistently restricted to the regions adjacent to the ventricles. Bar  = 15 μm B, E, H: BLBP protein in the brain of the Japanese eel as revealed by immunohistochemistry on the same sections than in A, D and G. One can see that immunoreactive cells have their nuclei along the ventricles and long processes running laterally. Bar  = 25 μm C, F, I: Merges showing overlapping (yellow color) of *cyp19a1b* mRNA and BLBP protein in the brain of the Japanese eel as revealed by immunohistochemistry on the same sections than in A, D and G. Only the radial processes do not show co-expression. Bar  = 20 μm.

**Figure 7:**
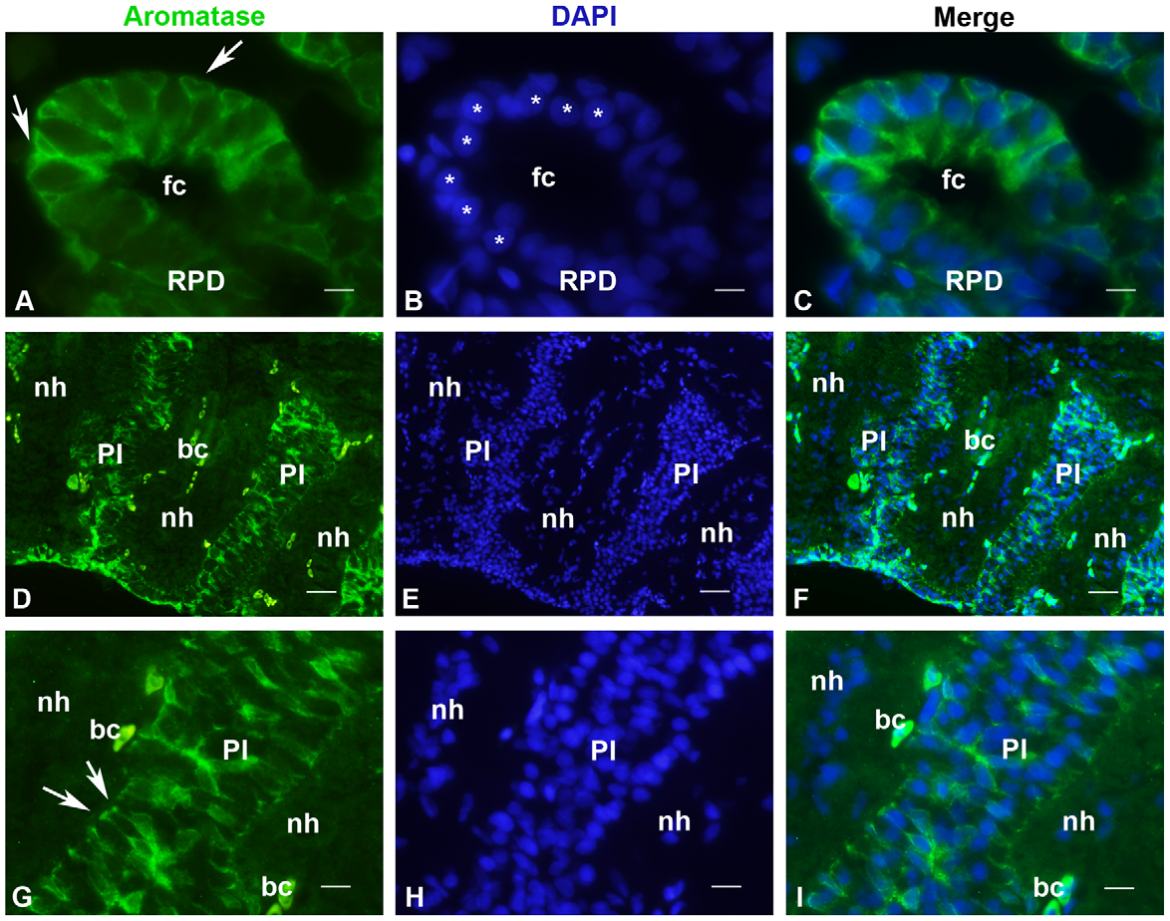
Expression of aromatase in the pituitary gland of the Japanese eel. A–C: High power view of aromatase-positive cells in the rostral pars distalis (RPD). One can see that positive cells are located in the outer region of the follicles and have a long process running to the follicle center (fc). Negative cells have their nuclei (stars) closer to the base of the follicles. Bar  = 7 μm D–F: Low power view of aromatase-positive cells in the pars intermedia (PI). Digitations of the neurohypophysis (nh) penetrate deeply within the proximal PI in which numerous positive cells are located. bc: blood cells showing endogenous fluorescence. Bar  = 75 μm G–H: High power view of aromatase-positive cells in the pars intermedia (PI) showing that positive cells have their nuclei located in the center of the PI cell population. However, they also have cytoplasmic processes ending onto the basal membrane that separates the neurohypophysis from the PI. bc: blood cells showing endogenous fluorescence. Bar  = 15 μm.

**Figure 8:**
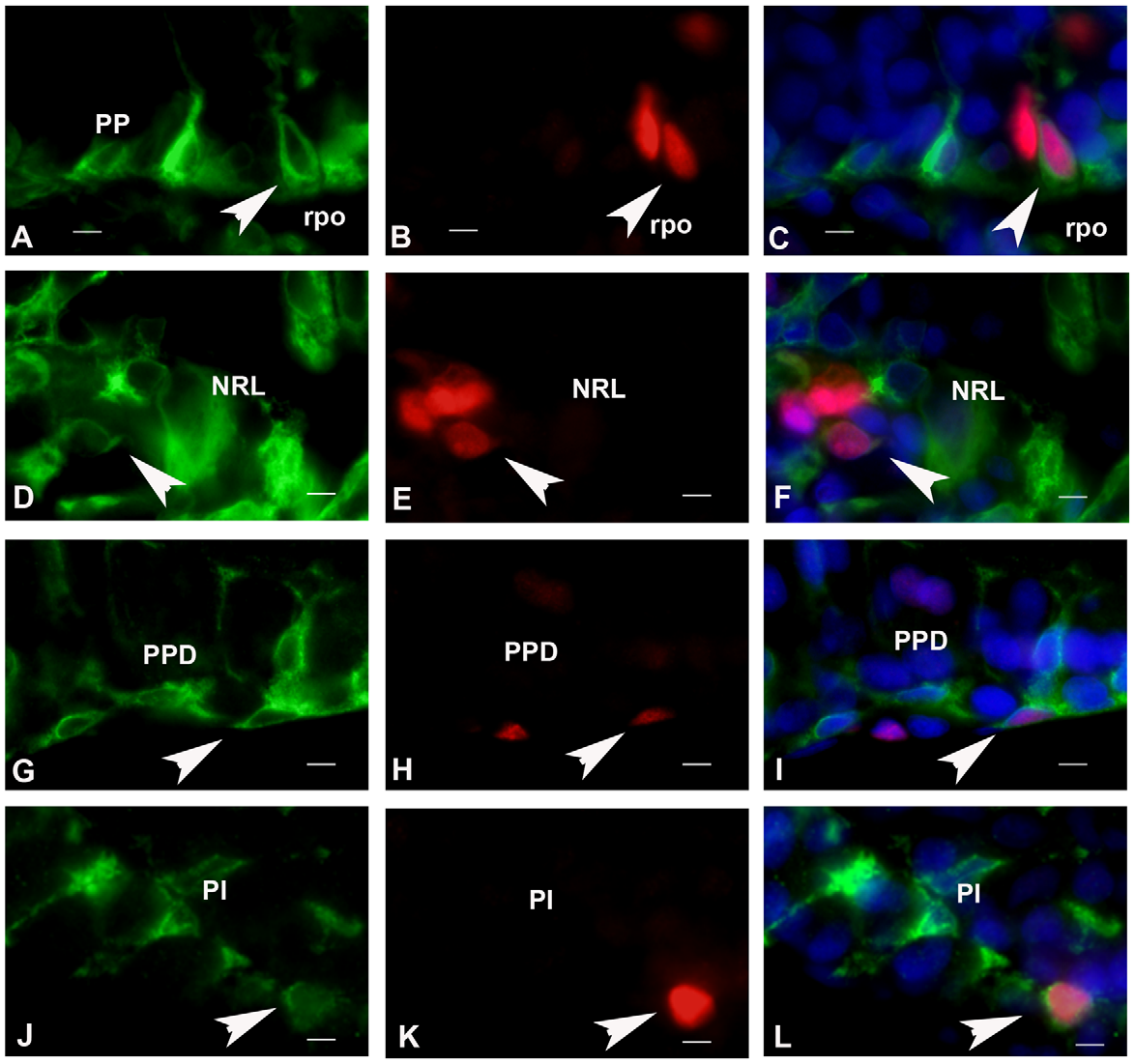
Aromatase-positive radial glial cells and pituitary cells exhibit proliferative activity. A–C: Aromatase-positive cell in the ventral periventricular preoptic nucleus (PP: arrow in A) exhibits a nucleus positive for the proliferation marker PCNA (arrows in B and C). rpo: preoptic recess. D–F: Aromatase-positive cell in the nucleus of the lateral recess (NRL; arrow in D) exhibits a nucleus positive for the proliferation marker PCNA (arrows in E and F). G–L: Two examples of aromatase/PCNA-positive cells the proximal pars distalis (PPD) and pars intermedia; arrows in G and J). Figures I and L show that the PCNA-positive nuclei (arrows in H and K) correspond to aromatase positive cells in G and J, respectively. All bars  = 5 μm.

**Figure 9:**
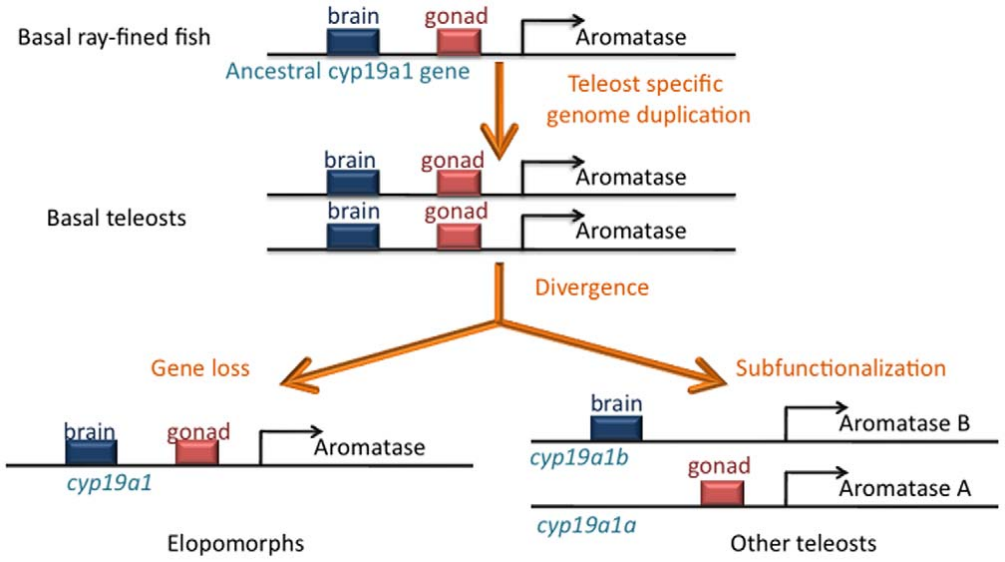
Hypothesis regarding evolution of the *cyp19a1* gene in the Actinopterygian lineage (ray-finned fish). From an ancestral gene having brain and gonad functions, the teleost specific genome duplication gave birth to two copies that evolved differently in Elopomorphs (Eels) and other teleosts. Soon after the duplication, eels probably lost one copy of the *cyp19a1* gene and this remaining copy retained brain and gonad functions. In other teleost fishes, a sufunctionalization process occurred that led to partition of functions between the two copies, *cyp19a1a* (gonad) and *cyp19a1b* (brain).

Cloning, quantitative-PCR and transcript analyses performed in Japanese and European eels suggested that eels have a single *cyp19a1* gene that would be expressed in both the brain and the gonads [32]–[34]. Phylogenetical analyses indicate that eel *cyp19a1* branches at the base of the teleost *cyp19a1* cluster, which is in agreement with the fact that the eel belongs to the Elopomorphs, a basal order of teleosts [32], [33]. The analysis of the current European eel draft genome [35] further supports the existence of a single *cyp19a1* gene (scaffold 1041.1) in this species. The uniqueness of the *cyp19a1* gene in eels would be intriguing given that recent studies evidenced the presence in both Japanese eel [36] and European eel genomes [35] of eight *hox* clusters, which result from the 3R event. The eels even have more *hox* clusters than other teleost species as all duplicated Hox clusters were conserved after the 3R, whereas crown teleosts lost one cluster (HoxCb or HoxDb) [36].

The above considerations suggest that the eels could have lost one copy of the duplicated *cyp19a1* gene resulting from the 3R event and thus that the *cyp19a1* situation in eels may reflect that of basal Actinopterygians. Therefore, the present study aimed at investigating whether expression and regulation of cyp19a1 in the brain of the Japanese eels is similar to that reported for *cyp19a1b* in other teleosts. The study was performed on unsexed immature eels and in males or females in which gametogenesis and steroidogenesis were stimulated by hormonal treatment. Indeed, only chronic treatments with fish (carp or salmon) pituitary homogenates can significantly induce ovarian development of eels [37], [38] as eels exhibit a striking life cycle with a blockade of sexual maturation at a prepubertal stage as long as their oceanic reproductive migration is prevented [39].

In the present study an antibody was generated against Japanese eel aromatase, allowing to study in details the expression and regulation of aromatase protein and *cyp19a1* mRNA. The results indicate that *cyp19a1* is, in the brain of the Japanese eel, only expressed in radial glial cells providing new insights into the evolution of aromatase expression and regulation in the Actinopterygian lineage.

## Materials and Methods

### Animals

Three-year-old female and male Japanese eels, *Anguilla japonica*, were obtained from a local fish farm in the middle of Taiwan and maintained in the aquatic facilities station of the National Kaohsiung Marine University (Taiwan, 22° N). Experimental fish were placed in an outdoor tank (2.5 cubic meters), under natural light and temperature conditions (water temperature range: 20–27°C). All procedures and investigations were approved by the College of Life Science of the National Taiwan Ocean University (Affidavit of Approval of Animal Use Protocol: N° 98029) and were performed in accordance with standard guiding principles.

### Treatment with fish pituitary extracts or human chorionic gonadotrophin

In the present study, we used either salmon pituitary homogenates (SPH: Shan Shui Technology Ltd., Kaohsiung, Taiwan) or human chorionic gonadotrophin (HCG: Gona-5000 injection, China Chemical & Pharmaceutical Co. Ltd., Taipei, Taiwan) to promote gonadal development in females and males, respectively. SPH treatment in females was performed as previously published [38]. Female eels were injected intraperitoneally (ip) with the homogenate of one salmon pituitary (20 mg dry weight) in 0.5 ml saline/fish. Animals were injected weekly for 8–12 weeks. Males were given one injection/week for 6 week of HCG (1 unit/gram body weight). Animals were sacrificed 4 days after the end of the hormonal treatment.

### Production of antibodies to Japanese eel aromatase

A synthetic 16-mer peptide (NH2-EKDSE LTMMF TPRRR Q- COOH) derived from the C terminus of the eel aromatase sequence (GenBank: AAS47028.1) was coupled on one end to bovine serum albumin and to keyhole limpet hemocyanin on the other end. This antigen was then used to produce a polyclonal antibody in rabbits. The antibody was prepared by ICON Biotechnology Co., Ltd (Taiwan).

### Protein extraction and western blotting

Pituitaries of Japanese eels were homogenized with a sonicator in modified RIPA (radioimmunoprecipitation assay) buffer (75 mM Tris-base, pH 7.4, 200 mM NaCl, 2 mM EDTA,0.1% SDS, 0.5% Na-deoxycholate and 1% NP-40 with a cocktail of protease inhibitors (Roche) and phenylmethylsulfonyl fluoride (PMSF)). The lysates were incubated on ice for 1 hour, and then centrifuged at 10,000 g for 20 minutes. The concentrations of the extracted proteins contained in the supernatants were measured using the Bio-Rad protein assay kit (Bio-Rad Co., Hercules, CA). 50 μg proteins were resolved on 12% SDS-PAGE and transferred onto a nitrocellulose (NC) membrane. After washing in TBST (150 mM NaCl, 20 mM Tris pH 7.6 and 0.1% Tween-20), nonspecific binding was blocked in TBST containing 5% skimmed milk powder for 1 hour. The blot was then incubated with the rabbit polyclonal antibody against Japanese eel aromatase (1∶1000) for 2 hours at room temperature. The blot was washed in TBST and incubated with the alkaline phosphatase-conjugated goat anti-rabbit IgG (1∶5000) (AnaSpec Inc., Fremont, CA) for 1 hour at room temperature. Finally, the protein was visualized using the BCIP/NBT liquid substrate system (Sigma).

### Antibody pre-absorption

Pre-absorption of the antibody with the antigen was performed to test the specificity of the immono-detection. 25 μM of synthetic 16-mer peptides derived from the C terminus of the eel aromatase sequence were incubated with the rabbit polyclonal antibody against Japanese eel aromatase (1∶1000) for 30 min at 37°C. This procedure led to the disappearance of the immunoreactivity in both western blottings and immunohistochemistry (see below).

### Immunohistochemistry (IHC)

The brains of Japanese eel were removed and fixed overnight in 4% paraformaldehyde diluted in 0.1 M sodium phosphate buffer with saline (PBS, pH 7.4). Paraffin transverse sections rehydrated through graded ethanol (100–30%), rinsed in PBS (pH 7.4) and then incubated in 3% H_2_O_2_ diluted with PBS for 10 minutes to block endogenous peroxidase activity. After washing in PBS containing 0.2% Triton, nonspecific binding was blocked in PBS containing 0.2% Triton and 1.5% skimmed milk powder. The sections were then incubated overnight at 4°C with the rabbit polyclonal antibody against eel aromatase (1∶500) or rabbit anti-BLBP (1∶500; brain lipid-binding protein, a marker of radial glial cells; Chemicon, Temecula, CA). Sections were washed three times in 0.2% Triton PBS and incubated with goat anti-rabbit Alexa fluor 488 (1∶200; Invitrogen Molecular Probes) for 1.5 hours at room temperature. Finally, sections were washed several times in PBS containing 0.2% Triton. The slides were mounted with Vectashield mounting medium containing 4′,6-diamidino-2-phenylindole (DAPI) (Vector Laboratories, Burlingame, CA) that permits visualization of cell nuclei.

For the double detection of aromatase and PCNA (Proliferating Cell Nuclear Antigen), sections were incubated with a mixture of rabbit antibodies to eel aromatase and mouse monoclonal antibodies to PCNA (1∶100; DAKO, Glostrup, Denmark; clone PC10). Slides were then exposed to a mixture of goat anti-rabbit Alexa fluor 488 (1∶200; Invitrogen Molecular Probes) or goat anti-mouse Alexa fluor 594 (1∶200; Invitrogen Molecular Probes).

### In situ hybridization (ISH) of cyp19a1 messengers

A partial *cyp19a1* cDNA sequence of 700 bp was cloned into the pGEM-T Easy Vector (Promega, Madison, WI) and linearized with *NcoI* or *SalI* to generate templates for synthesizing sense or antisense probes, respectively. Both sense and antisense riboprobes were synthesized with DIG RNA Labelling Mix (Roche, Indianapolis, IN) using T7 or SP6 RNA polymerases (Promega) by *in vitro* transcription. The brains of Japanese eel were removed and fixed overnight in 4% paraformaldehyde diluted in 0.1 M sodium phosphate buffer with saline (PBS, pH 7.4). The brains were dehydrated, embedded in paraffin and sectioned at 5 μm. Transverse sections were mounted onto TESPA (Sigma)-coated slides. The protocol for ISH was performed as previously described [40] with slight modifications. Paraffin sections were deparaffinized with xylene and rehydrated through a series of graded ethanol (100-30%). Sections were then washed with 0.85% NaCl and PBS before postfixation in 4% paraformaldehyde for 20 min. After washing in PBS, sections were treated for 5 min at 37°C with proteinase K (2 μg/ml) diluted in PBS, and fixed in 4% paraformaldehyde for 15 min. Sections were rinsed twice in 2× standard saline citrate (SSC). Hybridization was performed at 65°C overnight in a humidified chamber using 100 μl hybridization buffer (50% deionized formamide; 2× SSC; 5× Denhardt's solution; 50 μg/ml of yeast tRNA; 4 mM EDTA; 2.5%; dextran sulfate) containing the DIG-labeled probe (3 μg/ml). After hybridization, slides were washed in 2× SSC at 65°C (2×30 min), 2× SSC/50% formamide at 65°C (2×30 min), 0.2× SSC (1×15 min) and 0.1× SSC (1×15 min) at room temperature. Slides were next washed in 100 mM Tris-HCl (pH 7.5) containing 150 mM NaCl for 10 min and next washed in the same buffer containing 0.1% Triton and 0.5% of skimmed milk powder (2×30 min), and then incubated overnight at room temperature with anti-digoxigenin alkaline phosphatase Fab fragments (1∶2,000; Roche Pharma, Boulogne-Billancourt, France). On the next day, slides were incubated for 3–4 hours with an HNPP (2-hydroxy-3-naphtoic acid -2′-phenylanilide phosphate)/FastRED detection kit (Roche Pharma), according to the manufacturer's instructions.

### Microscopy and nomenclature for eel brain nuclei

Sections were observed under an epifluorescence microscope (Olympus Provis) equipped with a DP71 digital camera. Images were processed with the Olympus Analysis Cell software. Plates were assembled using Photoshop 7.0.1. In order to obtain reference sections, the brains of three-year-old female eels were prepared for routine histology and transversally sectioned at 5 μm, before staining with hematoxylin-eosin. The nomenclature used is that developed in the Japanese eel [41] with minor modifications.

## Results

### Specificity of the Japanese eel aromatase antibody

Figure 1 shows that western blotting of pituitary extracts using the Japanese eel aromatase antibody yielded a single band of the expected size (56 Kd). This band disappeared following pre-absorption with the peptide that was used to generate the antibodies. Similarly, the signal generated by the aromatase antibody on eel brain sections disappeared when the pre-absorbed antibody was used (data not shown).

These data and the fact that aromatase mRNA and protein were co-expressed in the same brain cells (see below) strongly established the specificity of the Japanese eel aromatase antiserum.

T*reatments with salmon pituitary extracts (females) or human chorionic gonadotrophin (males) increase aromatase immunoreactivity in the brain of Japanese eels.*

Our previous studies indicated that brain aromatase enzymatic activity and *cyp19a1* transcript levels were significantly higher in female eels treated with fish pituitary homogenates to induce experimental ovarian development [32], [38]. In order to increase the aromatase protein content and facilitate the characterization of the Japanese eel aromatase antibody, females and males were transferred to seawater and treated with salmon pituitary homogenates and human chorionic gonadotrophin, respectively. Figure 2 shows that such treatments strongly up-regulated aromatase-immunoreactivity in both males (Figures 2A–F) and females (Figures 2G–L).

In order to further assess the specificity of the aromatase immunoreactivity, transverse sections of female treated fish were stained for *cyp19a1* mRNA *in situ* hybridization together with aromatase immunohistochemistry. Figures 3 and 4 show that there was a very good correspondence between the distribution of the *cyp19a1* messengers and that of the aromatase protein.

While the *in situ* hybridization signal is restricted to cell soma lining the ventricles (Figures 3A–G and 4A–G), the aromatase antibodies label the same cell soma and long radial processes obviously corresponding to radial glial cells (Figures 3B–H and 4B–H). Parallel sections were always hybridized with the sense probes yielding absolutely no signal (data not shown).

In females treated with SPH, *cyp19a1* mRNA and aromatase-immunoreactivity were observed in periventricular regions of the forebrain (Figure 3 and 4A–F), notably in the ventral telencephalon, preoptic area and hypothalamus. A lower but consistent hybridization signal was found around the tectal and the fourth ventricles (Figures 4D–I). No mRNA was detected in the olfactory bulb, the cerebellum or the medulla oblongata.

In the telencephalon, the supracommissural nucleus of the subpallium (Vs) exhibited an intense signal (Figures 3A–C), while the dorsal, medial and lateral extents of the pallial regions showed little or no staining. In the diencephalon, the parvocellular preoptic nucleus (PP) and the magnocellular preoptic nucleus (PM) of preoptic area exhibited a very strong hybridization signal and numerous aromatase-positive cells (Figures 3D–I and 4A–C). Positive cells were also abundant in the central posterior thalamic nucleus (CP; Figures 4D–F) and periventricular nucleus of the posterior tuberculum. In the hypothalamus, *cyp19a1*mRNA-expressing and aromatase-positive cells were detected in most periventricular regions of the mediobasal hypothalamus surrounding the third ventricle, but also around the lateral recess.

### Aromatase is expressed in radial glial cells in the brain of adult Japanese eel

The morphology of the aromatase-positive cells clearly indicates that these cells are radial glial cells. They have a short proximal process in contact with the brain ventricles (Figure 5A) and a long distal radial process crossing the entire brain parenchyma (Figure 5B). These processes terminate by end-feet at the brain periphery (Figure 5C). To further confirm the radial glial nature of aromatase positive cells, we performed *in situ* hybridization for *cyp19a1* mRNA followed by BLBP (Brain Lipid Binding Protein) immunohistochemistry. As shown on Figure 6 the distribution of c*yp19a1*mRNA-containing cells was strictly identical to BLBP-positive cells, except that BLBP immunoreactivity was also observed in radial processes.

### Distribution of aromatase in the pituitary of adult Japanese eel

Abundant *cyp19a1* mRNAs were detected in cells of the pars distalis of the pituitary (data not shown), confirming previous data based on quantitative-PCR [32]. Expression of aromatase in the pituitary was confirmed by immunohistochemistry in males treated with HCG. Numerous cells could be detected in follicles of the rostral pars distalis (Figures 7A–C), proximal pars distalis (data not shown), and pars intermedia (Figures 7D–I). Figures 7A–C show that only cells with small nuclei located at the periphery of the follicles are stained. Such cells exhibited distal positive processes terminating by end-feet at the center of the follicles. In contrast, cells whose nuclei were larger and locate more centrally (Figure 7B) were unstained and likely correspond to prolactin cells.

Positive cells were also abundant in the pars intermedia, proximal pars distalis and pars intermedia. Figures 7D–F show digitations of the neurohypophysis penetrating deeply into the pars intermedia where many cells were stained by the aromatase antibody. Similar to those of the RPD, positive cells exhibited a long cytoplasmic process that end onto the basement membrane separating the pars intermedia from the neurohypophysis (Figures 7G–I).

### Aromatase-positive cells have proliferative activity in the brain and pituitary of adult Japanese eel

Double stainings for aromatase and PCNA were performed on the same sections. The results showed that a small proportion of the PCNA-positive cells were also aromatase positive (Figures 8A–F). Similarly, part of the aromatase positive cells in the different lobes of the pituitary exhibited a nucleus positive for PCNA (Figures 8G–L).

## Discussion

The present study is the first documenting the distribution of *cyp19a1* mRNA and protein in the brain of a basal teleost, the Japanese eel belonging to the order Elopomorph. The data show that, expression and regulation in the brain of Japanese eel appear similar to those reported in fish having a brain specific *cyp19a1b* gene.

### Aromatase expression is up-regulated by sex steroids

In addition to western blotting and routine absorption test, the specificity of the antibody against Japanese eel aromatase is demonstrated by the perfect overlapping with the *cyp19a1* messengers. Because *cyp19a1* expression is low in control eels, we used hormonal treatment to increase the expression levels. In both European and Japanese eels, it is known that the absence of sexual maturation is due to the lack of gonadotrophin production/release. Treatment with fish pituitary homogenates stimulates gametogenesis and steroidogenesis [37], [42], [43]. Among other effects, such treatments promote the positive feedback of sex steroids at the brain and pituitary levels. In agreement with the fact that sexual steroids increase aromatase activity and expression in the brain of fish [31], [44], notably in the Japanese eel [32], this study shows that SPH in females and HCG in males strongly up-regulate aromatase immunoreactivity in the brain. In some fish, it is known that estradiol directly promotes *cyp19a1b* expression in the brain due to the presence of a functional ERE in the proximal promoter of the gene [17], [21], [30], [31]. Previous studies based on aromatase activity and quantitative PCR [32], [38], indicated that expression of messengers and protein was highest in the forebrain, which is in agreement with the present study showing a high number of aromatase-positive cells in the subpallium, the preoptic area, the thalamus and hypothalamus, and lower immunoreactivity in the mesencephalon. This distribution is also in agreement with what was observed in other species [17], [19]–[22], [45]–[47].

### Aromatase expression is limited to radial glial cells of the brain

Thanks to the new antibody to Japanese eel aromatase, this study shows that aromatase expression is limited to radial glial cells, as observed in several other teleosts species. This unique characteristic was first discovered in the plainfin midshipman [19] and then in several other teleost species [17], [20]–[22], [26], [46], [48]–[52]. In mammals and birds, aromatase expression was mainly reported in neurons [53]–[56] and more recently in astrocytes [57], [58]. In zebra finch, expression of aromatase in radial glial cells was only shown after brain injury [59].

In the present study, the identification of aromatase-expressing cells is based on the radial morphology, the presence of a short proximal process to the ventricle and that of a long distal process terminating by en-feet at the brain surface. These characteristics already qualify the positive cells as *bona fide* radial glial cells [60]–[64]. Additionally, we show here that *cyp19a1*-expressing cells are also positive for BLBP, an established marker of radial glia in mammals [65], [66] and fish [25], [46], [67].

### Aromatase positive cells in the pituitary of the Japanese eel

Similar to that of some other fish [20], [47], [68], the Japanese eel pituitary exhibits higher aromatase activity compared to brain regions [38]. In the present study, we demonstrate that aromatase-positive cells are present in all lobes of the pituitary, which excludes that such cells correspond to a unique secretory cell type. The morphology of these cells that consistently exhibit a small nucleus and long cytoplasmic processes rather suggests that they correspond to agranular cells (folliculostellate cells) that have been reported to be abundant in the pituitary of European eels treated with estradiol [69]. Such agranular cells are notably reported at the periphery of the prolactin follicles. Similar to the aromatase positive-cells observed in the rostral pars distalis, these agranular cells have a small nucleus at the periphery of the follicle and a long proximal process to the follicle center [69]. Although the identification of these aromatase-positive cells as folliculostellate cells will have to be further documented, it is interesting to mention here that such cells express markers of radial glia such as protein S100ß, glial fibrillary acid protein (GFAP) and vimentin [70], [71], indicating that they share the same neuroectodermal origin than radial glia. Thus, aromatase could be another marker expressed by both radial glial cells and folliculostellate cells, at least in teleosts.

### Aromatase-positive cells exhibit proliferative activity

Some recent studies in fish have documented the fact that radial glial cells in adults are progenitor cells that generate neurons in developing and adult fish [17], [25], [26], [49], [72], [73], therefore sustaining the constant growth of the brain throughout life [74], [75]. As already documented in zebrafish and pejerrey [22], [26], aromatase-positive radial glial cells exhibit proliferative activity in eel. Although not demonstrated here, it is likely that at least part of the newborn cells give birth to new neurons. The reason why aromatase is expressed in such cells is still uncovered and remains an important question that has been discussed previously [17], [76], [77]. An increasing number of studies report that estrogens affect neurogenesis in a number of different models, but the results are conflicting [78]–[85]. Recent data in zebrafish indicate that estrogens would inhibit rather than stimulate radial glial cell proliferation [86]. Clearly, this question will require further studies in Japanese eels and other teleosts. Future studies should notably aim at further investigating the steroidogenic capacity of the eel brain and pituitary by investigating the expression of other key-steroidogenic.

### Evolutive considerations

The high aromatase activity, the expression of the *cyp19a1b* gene restricted to radial glial progenitors, and its extreme sensitivity to estrogens makes the situation in fish quite different from that known in mammals and birds. Thus, until now, it was assumed that the characteristics of aromatase expression in the brain of fish were the consequences of the teleost specific whole genome duplication (3R), which would have permitted independent evolution of the duplicated *cyp19a1* genes. In that hypothesis, a process of subfunctionalization followed by gain of new functions would have conferred new properties to the brain specific *cyp19a1b* gene.

However, the present study suggests that the ancestor of the *cyp19a1* gene in basal Actinopterygian fishes already had such characteristics. Indeed, there is strong evidence to suggest that the genome of the Japanese eel and European eels were duplicated. This is based on the fact that the eel genomes contain 8 hox clusters, indicating that it has retained the full repertoire of *hox* genes that resulted from the teleost specific genome duplication [35], [36]. Several studies documented the fact that many teleosts only have 7 hox clusters suggesting that they lost one of them after the 3R [5], [87].

In contrast, pervious work [32], [33] and the present study strongly suggest that eels have only one *cyp19a1* gene indicating that the second copy was lost soon after the teleost specific genome duplication. Therefore, one can hypothesize that the eel *cyp19a1* gene conserved characteristics of the ancestral Actinopterygian *cyp19a1* gene before the duplication (Figure 9). After the 3R, basal teleosts were equipped with two copies that underwent divergent fates in different teleost orders. While the single copy of the *cyp19a1* gene in Elopomorphs conserved the original brain and gonadal functions, in other fish a process known as subfunctionalization most likely occurred. According to this theory, the two copies are affected by deleterious mutations that differentially affect the subfunctions of the ancestral gene. As both copies are indispensable to carry out the function of the ancestral gene, the duplicated loci remain preserved through subfunctionalization [88], [89]. This process according to which two duplicated genes share the functions of their ancestor is also know as partition of functions. Some authors believe that partition of functions and independent lineage-specific evolution of duplicated genes have contributed to lineage diversification during teleost evolution [90].

In conclusion, by generating a highly specific antibody to Japanese eel aromatase, this study was able to address the open question of the brain expression of aromatase in Japanese eel providing additional evidence that this species has a single *cyp19a1* gene. The data show that expression, regulation and probably functions of this gene in the brain of Japanese eels are similar to those reported in other teleosts having a brain specific *cyp19a1b* gene. This suggests that such characteristics were present before the divergence of Elopomorphs and before the teleost-specific 3R event. Investigations on aromatase in basal Actinopterygians, such as chondrosteans, would further document whether these features reflect properties of the *cyp19a1* gene of ancestral Actinopterygians.
